# Specific and direct modulation of the interaction between adhesion GPCR GPR56/ADGRG1 and tissue transglutaminase 2 using synthetic ligands

**DOI:** 10.1038/s41598-020-74044-6

**Published:** 2020-10-09

**Authors:** Gabriel S. Salzman, Shu Zhang, Celia G. Fernandez, Demet Araç, Shohei Koide

**Affiliations:** 1 Biophysical Sciences Program, The University of Chicago, Chicago, IL 60637 USA; 2 Medical Scientist Training Program, The University of Chicago, Chicago, IL 60637 USA; 3 Department of Biochemistry and Molecular Biology, The University of Chicago, Chicago, IL 60637 USA; 4 Present Address: Translational Investigator Program, Department of Medicine, Stanford University, Palo Alto, CA 94304 USA; 5 Grossman Institute for Neuroscience, Quantitative Biology and Human Behavior, The University of Chicago, Chicago, IL 60637 USA; 6 Perlmutter Cancer Center, New York University Langone Health, New York, NY 10016 USA; 7 Department of Biochemistry and Molecular Pharmacology, New York University School of Medicine, New York, NY 10016 USA

**Keywords:** Biochemistry, Biophysics, Drug discovery, Neuroscience

## Abstract

Blocking the interaction between cell-surface receptors and their ligands is a proven therapeutic strategy. Adhesion G protein-coupled receptors (aGPCRs) are key cell-surface receptors that regulate numerous pathophysiological processes, and their large extracellular regions (ECRs) mediate ligand binding and function. The aGPCR GPR56/ADGRG1 regulates central nervous system myelination and melanoma progression by interacting with its ligand, tissue transglutaminase 2 (TG2), but the molecular basis for this interaction is largely undefined. Here, we show that the C-terminal portion of TG2 directly interacted with the GPR56 ECR with high-nanomolar affinity, and used site-directed mutagenesis to identify a patch of conserved residues on the pentraxin/laminin-neurexin-sex-hormone-binding-globulin-like (PLL) domain of GPR56 as the TG2 binding site. Importantly, we also show that the GPR56-TG2 interaction was blocked by previously-reported synthetic proteins, termed monobodies, that bind the GPR56 ECR in a domain- and species-specific manner. This work provides unique tools to modulate aGPCR-ligand binding and establishes a foundation for the development of aGPCR-targeted therapeutics.

## Introduction

Cell-surface receptors communicate signals across the plasma membrane. Vital to this process is the extracellular interaction between a signaling molecule and its receptor, which initiates a multi-step cascade, and ultimately results in a cellular response. Aberrant signaling can have catastrophic consequences, prompting the development of therapeutics to block the interactions between signaling ligands and their receptors^1–4^. For example, the programmed death-1 (PD-1) receptor on T-cells is the target of pembrolizumab and nivolumab, both cancer immunotherapeutic monoclonal antibodies that block the aberrant activation of PD-1, and thereby potentiate the immune system’s ability to attack cancer cells^5^. Though blocking receptor-ligand interactions has been successful in multiple contexts, developing blocking agents that target a specific receptor, or its specific ligand-binding domains, remains a nontrivial obstacle.

Adhesion G protein-coupled receptors (aGPCR) are cell-surface receptors that are involved in key pathophysiological processes including neurodevelopment, immunology, and tumorigenesis^6–9^. In addition to their seven-pass transmembrane helix bundle (7TM) that transduces signals across the plasma membrane^10^, aGPCRs characteristically contain large extracellular regions (ECRs), which comprise multiple adhesion domains involved in ligand binding^6^. Recent work has also shown that aGPCR ECRs play a direct role in the regulation of receptor function and downstream signaling^11–19^. Though most aGPCRs are still orphan receptors, the ligands of several aGPCRs have been identified as other cell-surface proteins or soluble extracellular matrix (ECM) proteins^14,20–22^. However, mechanistic studies of aGPCR-ligand interactions to identify binding sites and binding affinity have not yet followed the identification of these structurally complex protein ligands. Furthermore, strategies for modulating these interactions remain untested.

GPR56/ADGRG1 belongs to the aGPCR family and plays a critical role in diverse pathophysiological processes such as brain development^12,23–27^, major depressive disorder^28^, peripheral immunity^29,30^, CNS immunity^31^, skeletal muscle development^32–34^, pancreatic beta-cell function^35,36^, and cancer progression^37–43^. We have shown that the GPR56 ECR is composed of an N-terminal pentraxin and laminin/neurexin/sex hormone-binding globulin (PLL) domain and a juxtamembrane GPCR autoproteolysis-inducing (GAIN) domain and that the ECR plays a receptor-autonomous regulatory role in G protein signaling^12^. Furthermore, we have developed synthetic proteins, called monobodies, that bind to the GPR56 ECR and modulate G protein signaling, thus acting as allosteric agonists and allosteric inverse agonists^13^. Additionally, we identified a conserved, surface-exposed patch of the PLL domain that is necessary for GPR56-mediated myelination in zebrafish^12^. Though we speculated that this patch may be important for native ligand binding, we had no definitive information regarding the identity of such a ligand nor tools to probe such an interaction.

Tissue transglutaminase 2 (TG2) was reported as a putative ligand for GPR56 using unbiased pull-down experiments in 2006^44^. Studies have also implicated TG2 in murine models of melanoma progression and suggested that GPR56 inhibits melanoma progression in a TG2-dependent manner^37^. Additionally, more recent work has elucidated a complex array of interactions between neuronal GPR56 and microglial TG2 in the proliferation of oligodendrocyte precursor cells, leading to myelination in the central nervous system (CNS)^27,45^. Thus, there is strong evidence to support the importance of the GPR56-TG2 interaction in normal physiology and disease processes. TG2 is an enzyme that plays an important role in cross-linking proteins in the extracellular matrix (ECM). It has four domains (D1-D4), the second of which (D2) catalyzes a Ca^2+^-dependent transamidation reaction that crosslinks proteins in the ECM^46,47^. TG2 canonically plays roles in apoptosis, cell–matrix interactions, and tissue stability, as well as in pathologies including autoimmune disorders, neurodegenerative conditions, and cancer^48,49^. Though the GPR56-TG2 interaction was first identified over a decade ago, neither the affinity of TG2 for GPR56 nor its specific binding site have been determined. In early experiments, the region of GPR56 later identified as the PLL domain was shown to be necessary for TG2 interaction^44^. However, as experiments were not performed with purified proteins, it remained unclear whether the GPR56-TG2 interaction was a direct, binary interaction that might be suitable for targeting by pharmaceutical agents.

In this study, we used purified proteins to biochemically determine that the GPR56-TG2 interaction is indeed direct with binding affinity in the high-nanomolar range. We then utilized structure-guided mutagenesis analysis to identify the TG2 binding interface on GPR56. The binding interface is centered around a histidine residue that is critical for the in vivo myelination function of GPR56. Finally, we demonstrated that PLL-binding monobodies specifically block TG2 binding, paving the way for pharmacological disruption of native ligand binding to aGPCRs.

## Results

### TG2 interacts directly with GPR56

**Figure 1:**
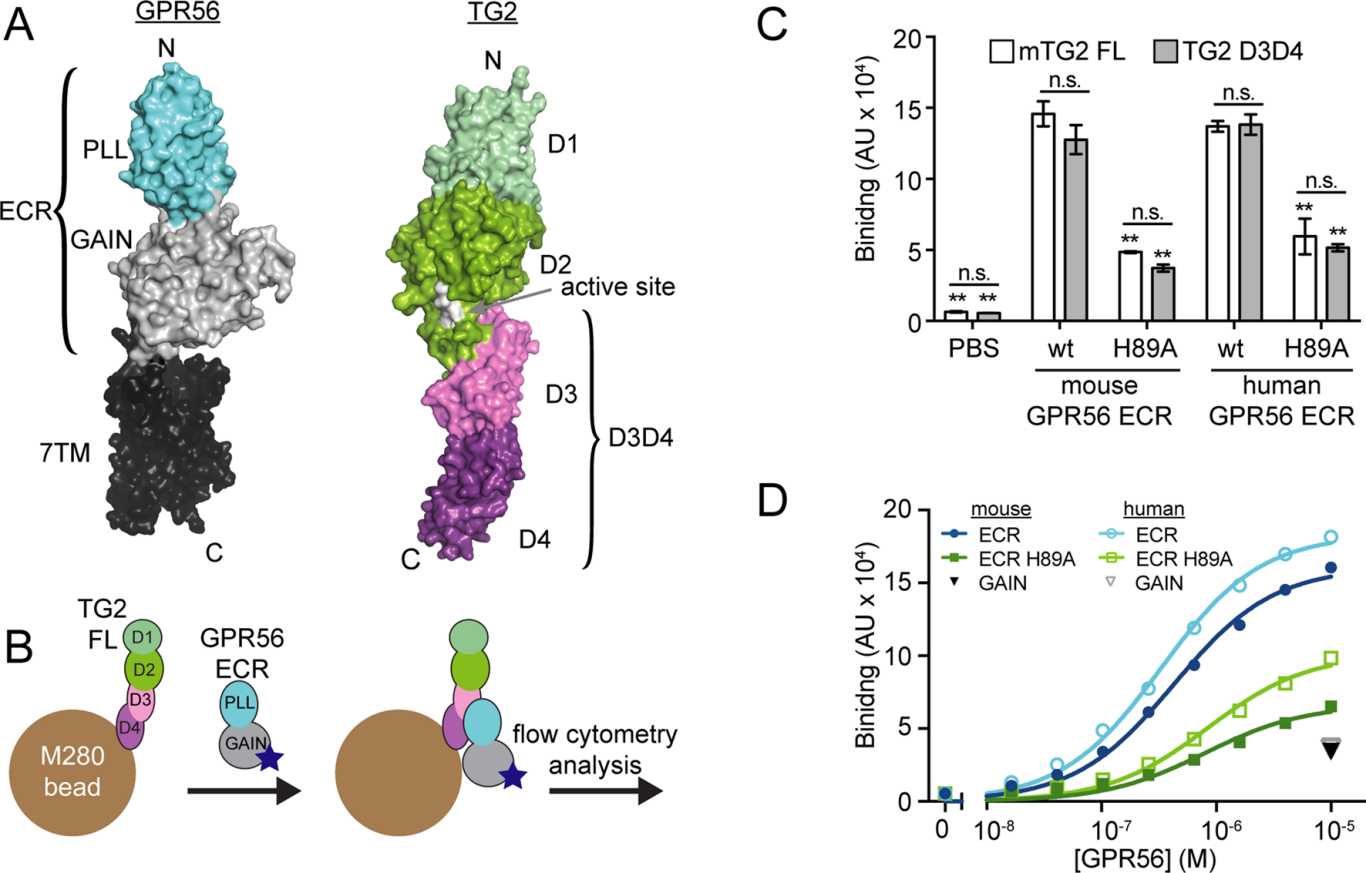
The GPR56-TG2 interaction is direct. (**A**) Structures of GPR56 (left) and TG2 (right, PDB: 2Q3Z) are colored by domain. The ECR of GPR56 (PDB: 5KVM) is shown in the context of a modelled 7TM based on the structure of the glucagon receptor (PDB: 46LR). (**B**) Schematic of a GPR56-TG2 binding assay between purified soluble GPR56 ECR and M280 beads coated with purified TG2 FL or TG2 D3D4. Blue star indicates fluorescent label. (**C**, **D**) GPR56-TG2 binding assays using setup outlined in (**B**). TG2 median fluorescence intensity (MFI) is plotted on the y-axis as TG2 binding signal. (**C**) Binding of TG2-coated beads to 100 nM GPR56 ECR, which was tetramerized^12,14^ to increase avidity. (**D**) Concentration titrations of monomeric GPR56 ECR constructs binding to TG2 D3D4-coated M280 beads. The H89A mutation was previously shown to confer a GPR56 loss-of-function phenotype in a zebrafish model^12^. GAIN domain is included as a negative control. Curves were fit to a simple one-to-one binding model to determine the dissociation constant, *K*_D_, of each interaction: mouse ECR, 440 ± 20 nM; human ECR, 330 ± 15 nM; mouse ECR H89A, 880 ± 80 nM; and human ECR H89A, 790 ± 11 nM. Error bars in (**C**) and (**D**) indicate S.E.M. of n = 3 independent measurements. Significance levels calculated by 2-way ANOVA with Bonferroni correction for multiple comparisons. Asterisks indicate comparison with wt mGPR56 ECR treated with FL mTG2. n.s., not significant; *p < 0.05; **p < 0.001. See Figures S1 and S5.

In order to determine if the interaction of TG2 with GPR56 is direct as opposed to one that requires another mediator, we first set out to obtain large quantities of purified proteins of high quality. Using a baculovirus-based system as previously described^11,12^, we expressed mouse and human GPR56 ECR constructs as well as mouse TG2 (mTG2) constructs in High Five insect cells. Our TG2 constructs corresponded to full-length mTG2 (mTG2 FL) and the C-terminal D3D4 domains (mTG2 D3D4; Fig. 1A), the region that was previously suggested to be sufficient to mediate GPR56-binding^44^. All GPR56 and TG2 constructs were cloned with an AVI-tag to facilitate biotinylation^50^. The proteins were purified using affinity chromatography followed by size-exclusion chromatography to ensure monodispersity. After purification, the samples were > 95% pure as judged using SDS-PAGE (Figure S1). We immobilized the biotinylated mTG2 constructs on streptavidin-coated M280 beads and measured binding to purified GPR56 constructs by flow cytometry analysis as previously described^13^ (Fig. 1B). Both purified mTG2 FL and purified mTG2 D3D4 bound strongly to purified wild-type (wt) human and mouse GPR56 ECRs (Fig. 1C), demonstrating that the GPR56-TG2 interaction is direct and confirming that the C-terminal pair of mTG2 domains (i.e. mTG2 D3D4) is sufficient to mediate GPR56 binding. As such, we used the mTG2 D3D4 construct for all binding experiments unless otherwise indicated.

### A loss-of-function mutation in the PLL domain decreases TG2 binding

We previously identified a conserved, surface-exposed patch on the GPR56 PLL domain and showed that mutating a highly-conserved residue therein to alanine (H89A) resulted in a GPR56 loss of function phenotype in a zebrafish model^12^. As this mutation did not affect cell-surface expression or basal activity of GPR56 in vitro, we hypothesized that this mutation within the conserved patch may have disrupted the interaction of GPR56 with a ligand. Indeed, the H89A mutant of GPR56 exhibited a significant decrease in binding to mTG2 (Fig. 1C), suggesting that the conserved patch of the PLL domain is involved in mTG2 binding.

We then quantified binding affinity of mTG2 for wt and H89A GPR56 constructs. Using the same experimental design described above, we found that mTG2 D3D4 bound to wt mouse and human GPR56 ECRs with apparent dissociation constant values (*K*_D_) of 440 ± 20 nM and 330 ± 15 nM, respectively (Fig. 1D), revealing that mTG2 D3D4 binds to mouse and human GPR56 with similar affinity. Consistent with the decreased binding signals in a single-point binding assay (Fig. 1C), the H89A mutation reduced the affinity of GPR56 with apparent *K*_D_ values of 880 ± 80 nM and 790 ± 11 nM for mouse and human GPR56 ECR H89A, respectively (Fig. 1D). mTG2 did not strongly interact with the human or mouse GAIN domain (Fig. 1D), further suggesting that the PLL domain mediates mTG2 binding^12,44^. Together, these results quantitatively define the thermodynamic parameters for the direct interaction between GPR56 and TG2 as well as the regions of each protein involved in the interaction.

### TG2 binds to a previously identified conserved patch on the PLL domain

**Figure 2:**
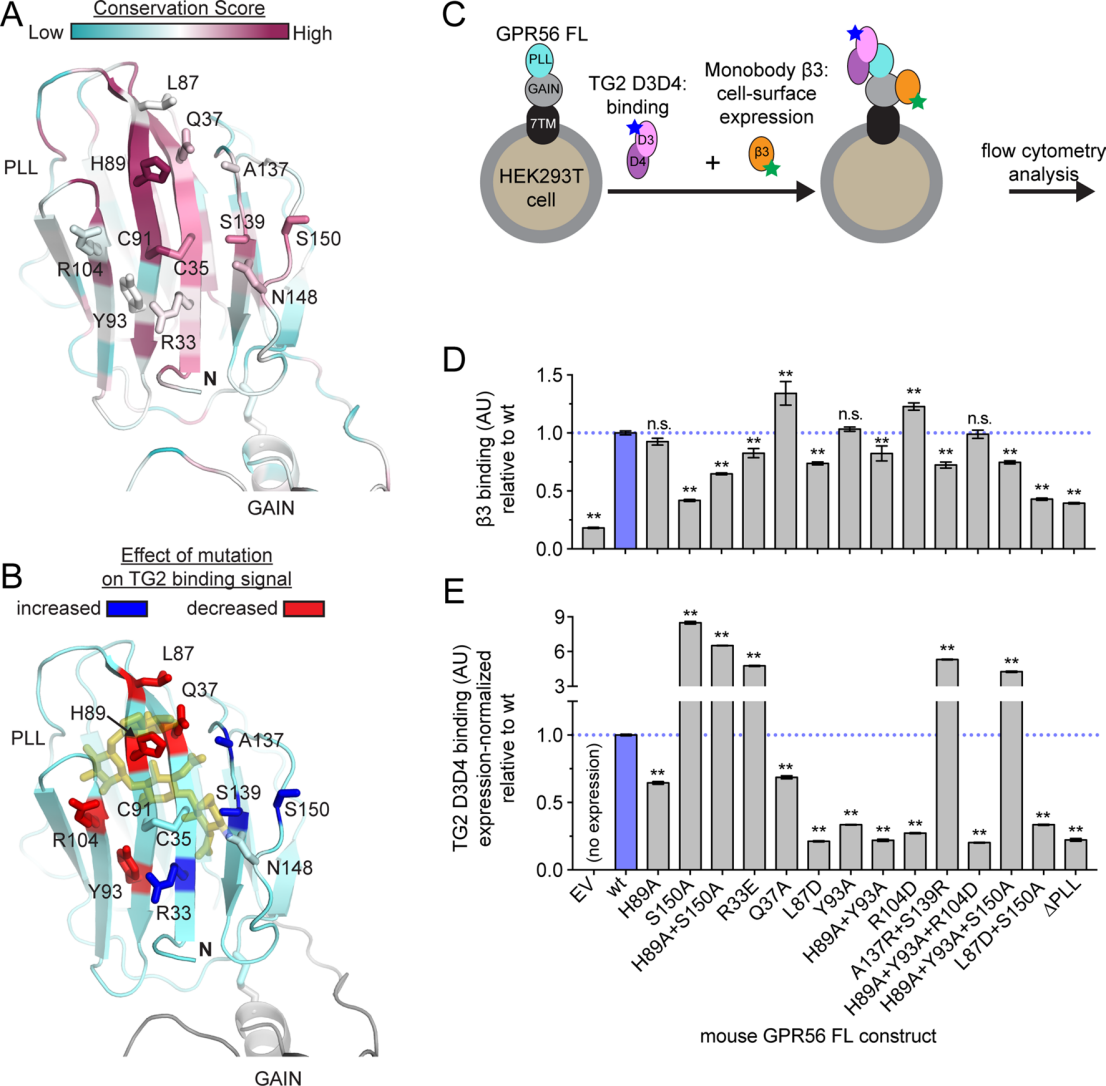
The conserved patch on the PLL domain mediates TG2 binding. (**A**, **B**) Cartoon of the conserved patch on the PLL domain (PDB: 5KVM). (**A**) Structure is colored by residue conservation score^51,52^. Figure adapted from reference^12^. (**B**) Sidechains are colored based on TG2 binding signal upon their mutation. The Asn148-linked glycan, which is expected to be removed with the S150A mutation, is shown as transparent yellow. (**C**) Schematic of the GPR56-TG2 binding assay using purified TG2 D3D4 and GPR56 FL constructs expressed on HEK293T cells. The binding signal of monobody β3, which binds the GAIN domain, was used to quantify GPR56 cell-surface expression. Green and blue stars represent different fluorescent labels. (**D**, **E**) Quantification of cell-surface expression of each mutant (**D**) and TG2 D3D4 binding signal (**E**) using the setup described in (**A**). Binding signal represents MFI. (**E**) Binding signal of 5 nM TG2 D3D4 (tetramerized) is measured within a defined range of cell-surface expression as detailed in Figure S4. Error bars in (**D**) and (**E**) indicate S.E.M. of n = 3 independent measurements. Significance levels calculated by 2-way ANOVA with Bonferroni correction for multiple comparisons. Asterisks indicate comparison with wt mGPR56. n.s., not significant; *p < 0.05; **p < 0.001. See Figures S2, S3, and S4.

In order to identify residues in the PLL domain that comprise the TG2 binding interface, we used bioinformatics and structural analyses to select residues with a high likelihood of involvement in protein–protein interaction. We started by examining our previously reported surface conservation analysis of GPR56 sequences from 150 species (Fig. 2A)^12,51,52^. We selected candidate residues based on their inter-species conservation (Figs. 2A and S2A), degree of surface exposure, and proximity to His89 (Fig. 2A,B). We also preferentially selected residues such as tyrosine and arginine that are generally enriched in protein–protein interaction interfaces^53^. Finally, as both mouse and human GPR56 robustly bound mTG2 (Fig. 1), we carried out pairwise surface conservation analysis to select residues conserved between mouse and human GPR56 (Figure S2B). In addition to His89, we chose seven residues (Arg33, Gln37, Leu87, Tyr93, Arg104, Ala137, and Ser139) for mutation analysis and constructed a panel of single, double, and triple mutations in the GPR56 FL construct.

To characterize the effects of GPR56 mutations on the GPR56-TG2 interaction in a more biologically relevant context, we developed an assay in which full-length GPR56 (GPR56 FL) constructs were expressed in HEK293T cells and binding of purified mTG2 D3D4 was quantified using flow cytometry (Fig. 2C). In this assay, mTG2 D3D4 bound strongly to human and mouse wt GPR56 FL displayed on the surface of mammalian cells (Figure S3A).

To utilize this assay to directly compare TG2 binding signal across our panel of GPR56 mutants, it was critical to account for differences in the cell-surface expression level between the GPR56 mutant constructs (Figure S4). Thus, we utilized a previously characterized monobody, Mb(hGPR56_β3), abbreviated β3, which binds the GAIN domains of mouse and human GPR56^13^ to quantify the surface expression level of GPR56. Then, we used the β3 staining to gate a subset of the cells that fell within a standardized narrow range of GPR56 surface expression, within which we quantified the degree of mTG2 D3D4 binding (i.e. expression-normalized TG2 D3D4 MFI; Figure S4). Using this method, we confirmed that the H89A mutation decreased mTG2 binding to both mouse and human GPR56 FL expressed on cells, and that PLL domain deletion (∆PLL) abolished mTG2 D3D4 binding (Figure S3B,C). Importantly, for each of the GPR56 mutant constructs we tested, if bulk mTG2 binding signal was increased or decreased versus wt, expression-normalized TG2 binding signal was also increased or decreased versus wt, respectively. These observations suggest that this expression-normalization protocol improves the precision of comparing mTG2 binding signal among mutant GPR56 FL constructs expressed on cell surface.

We identified a total of five PLL domain mutations (Q37A, L87D, H89A, Y93A, and R104D) that resulted in decreased mTG2 D3D4 binding signal (Figs. 2B,D,E and S3D). These residues are clustered in adjacent β-strands, as often seen for binding hot spot residues in protein–protein interaction^54^.

We found three mutants that substantially strengthened the interaction (R33E, A137R + S139R, and S150A) and form a contiguous surface (Fig. 2B,D,E). We designed the S150A mutation to disrupt the N-linked glycosylation consensus sequence, N148-X-S150^12^, so as to remove a previously identified N-linked glycan at Asn148 that is positioned immediately adjacent to His89 (Fig. 2B). S150A increased the affinity of the TG2-GPR56 interaction by approximately 40-fold, as measured using mTG2 D3D4-coated M280 beads and purified mouse GPR56 ECR S150A (Figure S5). Our results are consistent with the view that the presence of the Asn148-linked glycan weakens TG2 binding. Likewise, Arg33, Ala137, and Ser139 are located adjacent to the N148-linked glycan (Fig. 2B), and these mutations may perturb the location of the glycan. We note that we cannot exclude the possibility that these mutants directly affect TG2 binding and that further studies are needed to determine the mechanisms by which these mutations increase TG2 binding.

We found that the H89A + Y93A + R104D triple mutant completely abolished mTG2 binding without affecting cell-surface expression (Fig. 2D,E). It is a well-suited loss-of-function construct to use in future experiments focused on the GPR56-TG2 interaction, though it may affect the interactions between GPR56 and other ligands. All together, these results strongly suggest that the conserved patch of the PLL domain is the mTG2 binding site.

### PLL-binding monobodies specifically inhibit mTG2 binding to human or mouse GPR56

Though we were able to disrupt the GPR56-TG2 interaction by mutagenesis as discussed above, it would be ideal to be able to modulate this interaction in a biological system composed of endogenous, wt molecules using tools such as synthetic ligands. Such reagents may be valuable in future mechanistic and functional studies of the pathophysiology mediated by GPR56 and may even be considered lead molecules for therapeutic development. Specifically, we hypothesized that monobodies that bind the PLL domain of GPR56 may competitively block TG2 binding.

**Figure 3:**
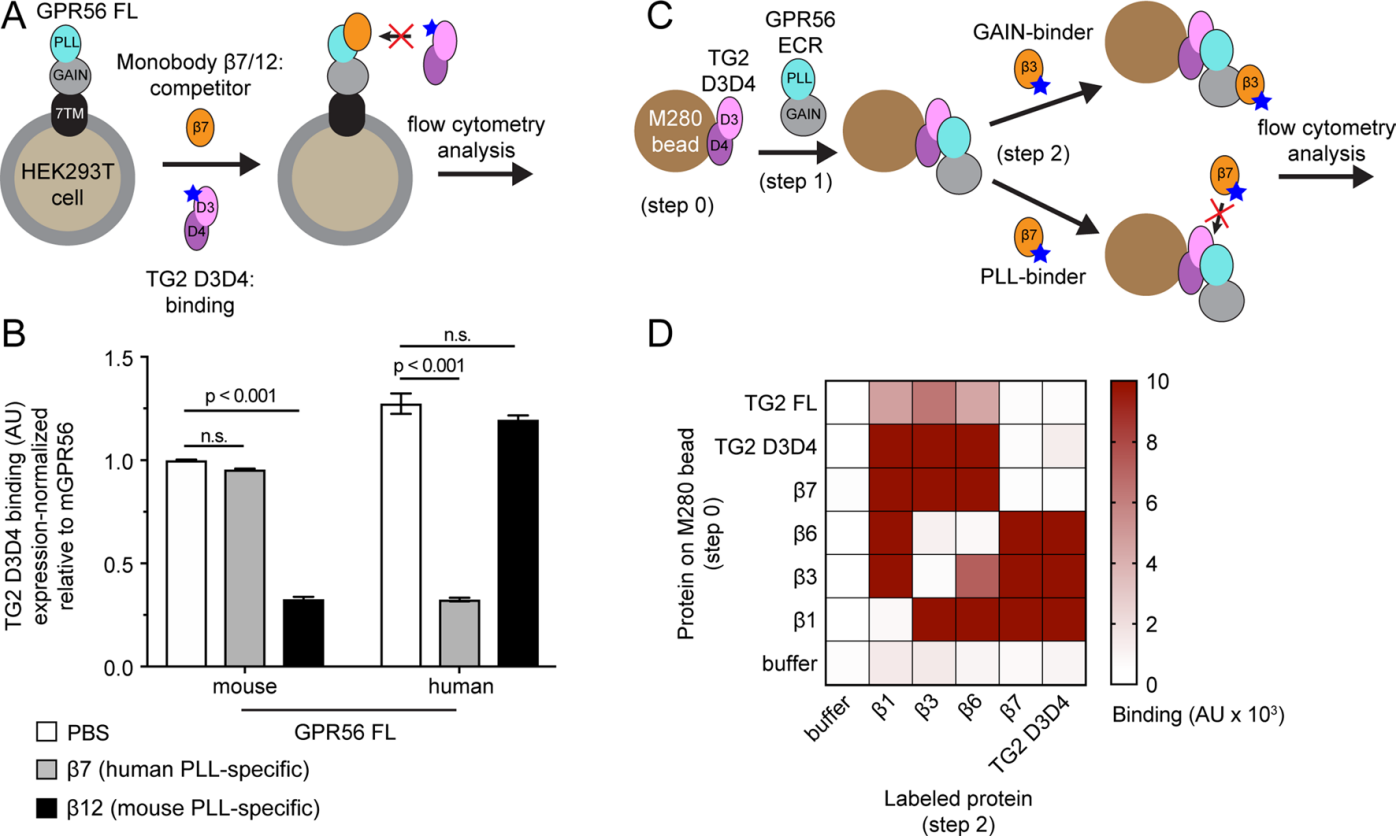
PLL-binding monobodies block TG2 binding. (**A**) Schematic of the monobody competition assay using GPR56 FL expressed on HEK293T cells and purified TG2 D3D4. Before and during incubation with labeled TG2 D3D4, cells were incubated with excess unlabeled monobody competitor (i.e. β7 or β12). Blue stars indicate fluorescent label. (**B**) HEK293T cells transfected with indicated GPR56 constructs were stained with purified TG2 D3D4 in the presence of excess unlabeled monobody competitor as described in (**A**). Binding signal represents MFI. Error bars indicate S.E.M. of n = 3 independent measurements. Significance levels shown were calculated by 2-way ANOVA with Bonferroni correction for multiple comparisons. n.s., not significant. (**C**) Schematic of the “sandwich” format binding assay: M280 beads coated with an immobilized protein (step 0) are incubated with unlabeled human GPR56 ECR (step 1) and then with a fluorescently labeled protein (step 2). By design, any protein pairs from steps 0 and 2 that that bind overlapping sites on hGPR56 ECR result in low binding signal. Conversely, protein pairs with non-overlapping binding sites on hGPR56 produce high binding signal. Blue stars indicate fluorescent label. (**D**) “Sandwich” format binding assay carried out as described in C. β1 binds the full ECR, while β3 and β6 bind the GAIN domain as previously described^13^. Binding signal represents MFI. See Figure S6.

Of the monobodies reported in our previous study^13^, Mb(hGPR56_β7), abbreviated β7, binds the human PLL domain but not the mouse counterpart. By contrast, Mb(mGPR56_β12), abbreviated β12, binds the mouse but not human PLL domain, supporting the utility of these two monobodies as excellent controls in our experiments (Figure S6A). In a competitive binding assay, β7 significantly decreased TG2 D3D4 binding to human GPR56, suggesting that β7 and mTG2 bind to overlapping epitopes on GPR56. As expected, β7 did not affect mTG2 D3D4 binding to mouse GPR56. Likewise, we observed that β12 inhibited mTG2 D3D4 binding to mouse GPR56 but not to human GPR56 (Figs. 3B and S6B). Thus, these data show that we have developed synthetic ligands that block ligand binding of human and mouse GPR56.

To confirm β7 and mTG2 are not able to simultaneously bind human GPR56, we carried out a “sandwich” binding assay. In order to test if a pair of proteins (e.g. TG2 and β7) compete with each other for binding to the GPR56 ECR, we immobilized the first protein of interest on M280 beads, incubated with unlabeled human GPR56 ECR, and stained with a fluorescently labeled version of the second protein of interest (Fig. 3C). In this format, a strong binding signal is observed when pairs of proteins are able to simultaneously interact with the GPR56 ECR. A weak binding signal is observed when pairs of proteins compete with each other for GPR56 binding (i.e. via competitive or allosteric inhibition). As expected, for each pair of identical proteins (shown on the diagonal), we observed weak binding signal. Weak binding was also seen for mTG2 FL and mTG2 D3D4, which we expected to interact with GPR56 via the same binding site. In this assay, the combination of β7 with mTG2 (both FL and D3D4) yielded a weak binding signal (Fig. 3D), supporting our earlier results that β7 and mTG2 are not able to simultaneously bind human GPR56. Monobody β3 was included in this experiment as a negative control, as it is known to bind the GAIN domain and not the PLL domain. As expected, the presence of β3 had no effect on mTG2 FL or mTG2 D3D4 binding signal. Finally, monobodies β1 and β6, which also bind the human GPR56 ECR (Figure S6A)^13^, did not affect TG2 binding signal, suggesting they do not interact with the same region of the PLL domain as TG2. These data strongly support that β7 blocks mTG2 binding to GPR56.

## Discussion

The interaction between GPR56 and TG2 has been implicated in remarkably diverse biological processes^27,34,37,43,44^. Thus, with the ultimate goal of modulating this interaction as a potential therapeutic strategy for multiple diseases, there remains great interest in advancing a fundamental biochemical understanding of GPR56-TG2 binding.

We first established a quantitative binding assay to measure the affinity of mTG2 for GPR56 in their native states. We found that mTG2 has similar affinity to human and mouse GPR56. The small difference (~ 1.3-fold change in *K*_D_) suggests a conserved interaction interface between TG2 and GPR56 in human and mouse. Though previous studies using immunoblotting have suggested that human GPR56 interacts more strongly with mTG2 than human TG2, these experiments do not necessarily reflect affinity of native proteins^55^. Future studies may elucidate the conservation of the interaction between GPR56 and human TG2.

**Figure 4:**
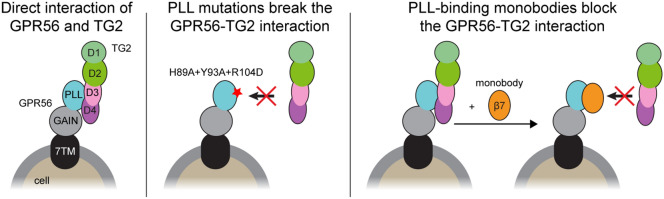
Modulation of the GPR56-TG2 interaction. Summary of the results presented are depicted. (Left) Depiction of the interaction between wt FL GPR56 and TG2. (Middle) Mutations to the PLL domain (red star) block mTG2 binding. (Right) PLL-binding monobodies disrupt the interaction between GPR56 and mTG2.

Our observation that mTG2 interacts with the previously identified surface-exposed conserved patch on the PLL domain of both human and mouse GPR56 was striking given our prior speculation that this region of the PLL domain mediates ligand binding^12^. Indeed, we found that the H89A mutation as well as several other nearby mutations decreased TG2 binding affinity (Figs. 1, 2, 4, S3, and S5). Thus, we speculate that the H89A-induced loss-of-function phenotype we previously observed in a zebrafish model of GPR56-mediated myelination^12^ is due, at least in part, to the abrogation of TG2 binding. This hypothesis is further supported by recently published work elucidating the roles played by GPR56 and TG2 in oligodendrocyte precursor cell proliferation and myelination^27^.

In addition to blocking TG2 binding with mutagenesis, we were able to disrupt binding in a system composed of wt GPR56 and TG2 with synthetic monobody ligands (Figs. 3, 4, and S6). These PLL-binding monobodies are notably specific in that they interact with a single domain in the GPR56 ECR and do not cross-react between the human and mouse receptor (Figure S6A). This specificity allows for precise functional dissection of the individual GPR56 domains. Additionally, we previously showed that monobodies β7 and β12 interact with human and mouse GPR56 ECR with *K*_D_ values of 66.7 nM and 27.9 nM, respectively, using surface plasmon resonance^13^. These *K*_D_ values are about an order of magnitude lower than the apparent *K*_D_ values we report for the GPR56-TG2 interaction (Fig. 1D). Thus, the affinity of these monobodies for GPR56 is likely high enough to out-compete TG2 in vivo. All together, these data serve as proof of concept that synthetic molecules may be used to block interactions between aGPCRs and their native ligands. Such a strategy may be used in the context of treating aGPCR-mediated pathologies with pharmaceutical agents.

TG2 is a ubiquitously expressed enzyme that plays many biological roles^56,57^. The catalytic activity of TG2 (i.e. protein crosslinking) is at the core of much of the mechanistic understanding of TG2 function. However, the active site of TG2 is not present in the GPR56-binding fragment of TG2 (Fig. 1). Thus, though it is clear that enzymatic activity is not required for GPR56 binding, the role of TG2-mediated crosslinking in the context of GPR56 binding remains unclear. More recent work has elucidated a complex array of interactions between GPR56, TG2, and laminin, another ECM protein, in the proliferation of oligodendrocyte precursor cells, leading to myelination in the central nervous system (CNS)^27,45^. However, these studies went on to show that, in the presence of TG2 and laminin, dissociation of the N-terminal fragment of GPR56 was observed, leading to robust *Stachel*-mediated activation of the receptor. Thus, future studies examining the enzymatic activity of TG2 in the context of GPR56 and laminin may shed light on this mechanism of receptor activation. Broadly, we anticipate that the tools presented in this manuscript, including monobodies and GPR56 point mutations that abrogate binding while preserving cell-surface expression, will facilitate functional studies dissecting complicated macromolecular assemblies involving GPR56, perhaps even in the context of native ECM, and thereby pave the way to developing GPR56-targeted pharmaceutical agents.

## Materials and methods

### Cloning, expression, and purification of TG2 fragments

Full length mTG2 was provided as kind gift from Lei Xu (University of Rochester). The TG2 FL and the TG2 D3D4 constructs (residues T471-A686) were cloned into the vector pVL1393. N-terminal 6xHIS and AVI-tags were added to each construct to facilitate purification and biotinylation, respectively. Baculoviruses were generated for cytosolic insect cell expression as previously described^11,12^. Large-scale High Five insect cell cultures were infected with baculovirus and grown for 48 h before the cell pellet was harvested by centrifugation and frozen at – 80 °C. Cell pellets were thawed and cells were lysed at 4 °C in a manual homogenizer in 10 mM HEPES pH 7.2 + 150 mM NaCl + 1 mM TCEP + 2 mM PMSF. The supernatant was collected by centrifugation (37,000 × g for 1 h) and incubated with Ni–NTA sepharose resin for 3–5 h at 4 °C with constant stirring. Proteins were biotinylated before eluting from resin as previously described^18^. Briefly, the beads were washed and incubated with purified BirA biotin ligase + biotin + ATP for 1 h at 27 °C in 50 mM Bicine pH 8.3 + 150 mM NaCl + 10 mM Mg acetate. The biotinylated TG2 was then eluted in 10 mM HEPES pH 7.2 + 150 mM NaCl + 1 mM TCEP + 200 mM imidazole. The eluent was filtered and injected into a Superdex 10/300 Gel Filtration column equilibrated in 10 mM HEPES pH 7.2 + 150 mM NaCl + 0.5 mM TCEP for TG2 FL and 10 mM HEPES pH 7.2 + 150 mM NaCl + 0.5 mM TCEP + 1 mM CaCl_2_ for TG2 D3D4. Peak fractions were pooled and frozen in liquid nitrogen.

### HEK 293T cell transfection

HEK293T cells were cultured in 6-well plates in Dulbecco’s modified Eagle’s medium (DMEM; GIBCO) supplemented with 10% FBS (Sigma), at 37 °C in 5% CO_2_. Transient transfection was performed with cells at 50–60% confluence as follows: 2 µg cDNA was diluted into 50 µL final volume of serum-free DMEM, while 3 µL LipoD293 transfection reagent (SignaGen) was added to 47 µL serum-free DMEM. Diluted LipoD293 was then added to diluted cDNA, and complexes were allowed to form by incubation at room temperature for 10 min. The transfection complex mixture was then added dropwise to each well. After 48 h incubation at 37 °C/5% CO_2_, the cells were detached using citric saline solution (135 mM KCl + 50 mM sodium citrate) and washed with PBS + 2% BSA.

### Flow cytometry

#### HEK293T-expressed GPR56 FL

HEK293T cells were transfected with wt or mutant GPR56 constructs as described above and co-stained with monobody β3 + neutravidin-488 tetramers and TG2 + neutravidin-650 tetramers. Tetramers were independently prepared in the excess of free biotin and soluble unlabeled neutravidin before mixing together to avoid the possibility of forming β3 + NAV650 and TG2 + NAV488 tetramers. To normalize for differential expression of GPR56 mutants, TG2 binding signal was normalized to a particular bin of β3 binding signal (Figure S4). Thus, only cells with similar expression of wt or mutant GPR56 FL were included in the analysis. Data were collected on an AccuriC6 flow cytometer and processed in FlowJo.

#### HEK293T-expressed GPR56; TG2 and monobody competition

HEK293T cells were transfected with GPR56 constructs (wt or mutant), incubated with 500-fold molar excess of unlabeled tetramerized monobody competitor (β7 or β12), and then co-stained with 500 nM monobody β3 + neutravidin-488 tetramers and 1 nM TG2 + neutravidin-650 tetramers. Binding signal was normalized to GPR56 expression as described above (Figure S4). Data were collected on an AccuriC6 flow cytometer and processed in FlowJo.

#### TG2-coated M280 beads and affinity measurement

M280 bead-binding assay was carried out as previously described^12^. In short, M280 beads were coated with TG2, following which, purified and biotinylated GPR56 fragments were incubated with beads at various concentrations. Neutravidin-650 was then added to detect the GPR56 fragments. Binding signal versus GPR56 fragment concentration was plotted to calculate the apparent dissociation constant, *K*_D_. Data were collected on an Intellicyt flow cytometer, initially processed in FlowJo, and standard 1-to-1 binding curve-fitting was done in Prism.

### Tissue culture

Insect cell culture and mammalian cell culture were both performed as previously described^18^. In short, Hi Five insect cells (Trichoplusia ni) were cultured in Insect-Xpress medium (Lonza) supplemented with 10 mg/mL gentamicin at 27 °C and were used for production of recombinant proteins. HEK293T mammalian cells (Homo sapiens) were cultured in Dulbecco’s modified Eagle’s medium (DMEM; GIBCO) supplemented with 10% FBS (Sigma) at 37 °C in 5% CO_2_ and were used for cell-surface expression assays and flow cytometry binding assays.

## Supplementary information

Supplementary information.
