# Supplementary material for: Specific and direct modulation of the interaction between adhesion GPCR GPR56/ADGRG1 and tissue transglutaminase 2 using synthetic ligands
Source: Sci Rep. 2020 Oct 9;10:16912. doi: 10.1038/s41598-020-74044-6 (PMC7547085; doi:10.1038/s41598-020-74044-6)
Supplement: Supplementary file 1 — Supplementary information. [file 41598_2020_74044_MOESM1_ESM.pdf]

Specific and direct modulation of the interaction between adhesion GPCR GPR56/ADGRG1 and tissue transglutaminase 2 using synthetic ligands

Gabriel S. Salzman, Shu Zhang, Celia G. Fernandez, Demet Araç, and Shohei Koide

Supplementary Information

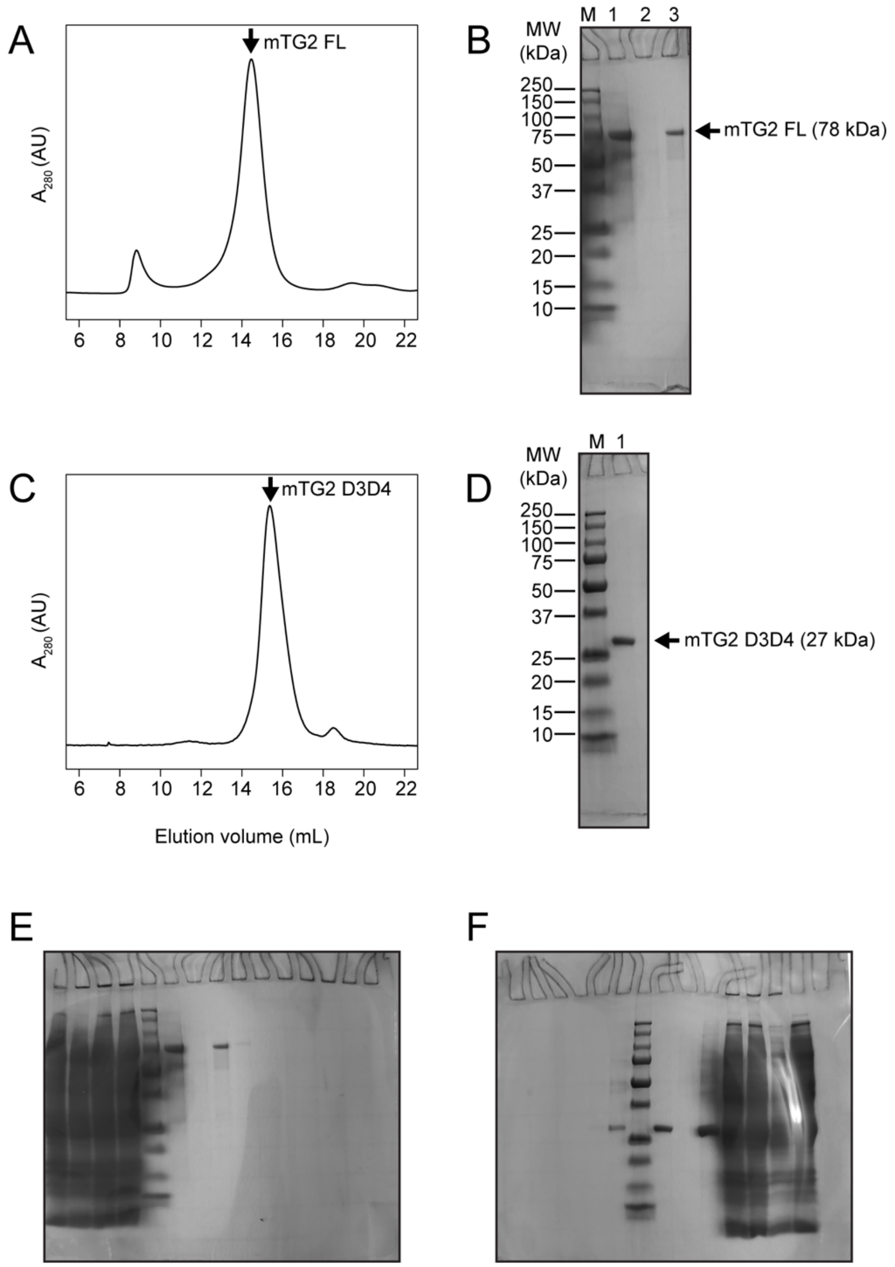

Salzman et al., Figure S1

Figure S1. Purification of TG2 constructs. (A) Following affinity chromatography, mTG2 FL was purified by gel filtration chromatography. Monodispersed peak fractions (arrow) were isolated. (B) SDS-PAGE of peak fractions from A showing purified mTG2 FL at the expected molecular weight (arrow). Lanes labeled as follows: M, marker; 1, irrelevant sample; 2, blank; 3, mTG2 FL. Uncropped gel shown in E. (C-D) Analogous purification of mTG2 D3D4. (D) Lanes labeled as follows: M, marker; 1, mTG2 D3D4. Uncropped gel shown in F. (E-F) Uncropped gels from B and D as above.

A

```
Conservation:         6  679999988888 766777786      7877676766767767766 6  6     676777799999899
bat                27 G-FREDFRFCGQRNQ-TQKSSLRYER-WTQLRISIENSEEALTVHAPFPGVPGAS----HFFPDPRGLYHFCLSW
bovine             26 G-PREDFRFCGQRNQ-TQNSSLHYKQ-ASQLHISIRNSEEALTIHAPFPGVQSAS----WPFPLPRGLYHFCLYW
chicken            19 G-RQEDFRFCGDRNQ-TENSSVIYEH-SS-ANISIENTAQALVIRSPFLADRTTPS-YQYSLPTTLGRYRFCIYW
chimp              27 G-HREDFRFCSQRNQ-THRSSLHYKP-TPDLRISIENSEEALTVHAPFPAAHPAS----QSFPDPRGLYHFCLYW
cuckoo             19 GDQEEDFRFCGDRNQ-TQNSSVIYEH-SP-ANISIENTAQALIIKRPFLPNRRSSY-YKYTLPPTLGRYRFCIYW
dolphin            26 G-PREDFRFCGQRNQ-TQNSSLHYKR-TSELHISVKNTEEALAVHAPFPGAHLAP----RSFPHPRGLYHFCLYW
elephant           26 G-LQEDFRFCGQRNQ-TQTSNLRYEQ-TAVLHISIKNSEGALTVHAPFPAAPGAS----RVLPDRRGLYHFCLYW
gorilla            27 G-HREDFRFCSQRNQ-THRSSLHYKP-TADLRISIENSEEALTVHAPFPAARPAS----QSFPDPRGLYHFCLYW
human              27 G-HREDFRFCSQRNQ-THRSSLHYKP-TPDLRISIENSEEALTVHAPFPAAHPAS----RSFPDPRGLYHFCLYW
monkey             27 G-HREDFRFCSQRNQ-THISSLHYKF-TPDLRISIENSEEALTVHAPFPEAHPAS----RSFPHPRGLYHFCLYW
mouse              27 S-PREDFRFCGQRNQ-TQQSTLHYDQ-SSEPHIFVWNTEETLTIRAPFLAAPDIP----RFFPEPRGLYHFCLYW
rat                27 S-PREDFRFCGQRNQ-TQQSTLHYDQ-TSEPHIFVWNTDESLTIRAPFPAAPDIP----YFFPEPRGLYHFCLYW
sperm_whale        26 G-PREDFRFCGQRNQ-TQNSGLHYKR-ASELHISIRNTEEALTVHAPFPGAHPAP----RSFPHPRGLYHFCLYW
zebrafish          26 DN-DRDFKMCGKWLHGIAPQNLEYDLKTGCERIEISANESTLSIQGRITAKCTQSSSIQLDSNPHQNQSHFCVFW

Conservation:         677 6 766  878777676 6   667666666    6       7667777779787666  6 86
bat                95 NRHAGELHLL-YGKNDFLLSDQASGLLCFQGQEP-ILAQGPRMLATSVSSWWRPQNTSLPSAAGFTFS
bovine             94 NRHAGKLHLR-YGKKDFVLSDQALDLLCFRHQEE-TLVPGPPLFATSVSSWWSPQNTSLPSAASFIFS
chicken            89 FKANRTLWLA-YGKKSFFLGHDPADGIARGLEKTK-----ASILNVS-YVFKGQKNTSLESASEYLFP
chimp              95 NRHAGRLHLL-YGKRDFLLSDKASSLLCFQHQEE-SLAQGPPLLATSVTSWWSPQNISLPSAASFTFS
cuckoo             90 FKANRTLRLV-YGKQSFLLGRDPSNSITQGKESQKTERTSTSIFNVS-YVLKGGKNTSLASASEYSFS
dolphin            94 NRHAGKLHLR-YGKSDFVLSNQASDLLCFRHQEE-SLAEGAPLFATSVSSWWSPQNTSLPSAAGFIFS
elephant           94 SRHTGKLHLR-YGKNDFLLSDQASGLLCFRHQEE-SLVQGPPLLATSVSSWWSPQNTSLPGATSFTFS
gorilla            95 NRHAGRLHLL-YGKHDFLLSDKASSLLCFQHQEE-SLAQGPPLLATSVTSWWSPQNVSLPSAASFTFS
human              95 NRHAGRLHLL-YGKRDFLLSDKASSLLCFQHQEE-SLAQGPPLLATSVTSWWSPQNISLPSAASFTFS
monkey             95 DRHAGRLHLL-YGKHDFLLSDQASSLLCFQHQEE-SLAQGPPLFATSVTSWWSPQNISLPSASNFTFS
mouse              95 SRHTGRLHLR-YGKHDYLLSSQASRLLCFQKQEQ-SLKQGAPLIATSVSSWQIPQNTSLPGAPSFIFS
rat                95 SRHTGRLHLR-YGKNDYLLSSRASNLLCYRKQEE-SLKQGAPLVATSVSSWQSPQNTSLPGAPSFIFS
sperm_whale        94 NRHAGKLHLR-YGKSDFVLSNQASDLLCFRRQEE-SLAEGAPLFATSVSSWWSPQNTSLPSAAGFIFS
zebrafish         100 EPLLDLLIVEVNGKNHTLCKPNGLQGTCCTDLSQ--GVQ-------DNAHMYGIVNGSVKGD--IITG
```

B

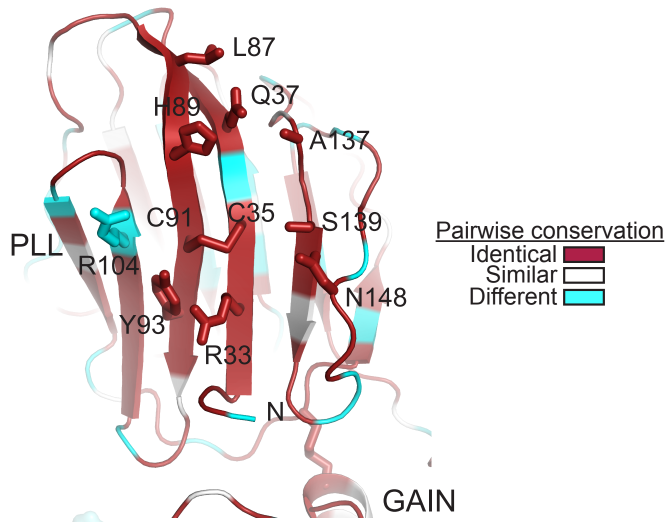

C

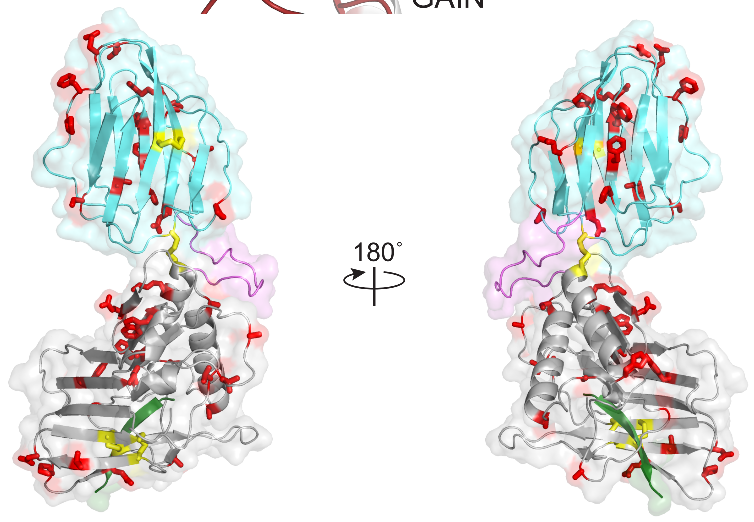

Salzman et al., Figure S2

Figure S2. Conservation of GPR56 residues involved in TG2 binding and cancer. (A) Multiple sequence alignment of GPR56 PLL domains from 14 different species. The sequence UniProt (UniParc) identification numbers are: bat, G1PD76; bovine, A4IF70; chicken, E1C0Q2; chimp, Q50DM7; cucoo, A0A091FQY3; dolphin, (UPI000441A997); elephant, G3T7E2; gorilla, Q50DM6; human, Q9Y653; monkey, (Q50DM8); mouse, Q8K209; rat, Q8K3V3; sperm whale, (UPI00042C4060); and zebrafish, F1QZM9. Relative entropy conservation score was calculated with the Protein Residue Conservation Prediction tool [1]. Residues highlighted in yellow are involved in the intra-PLL domain disulfide bond. Residues colored blue or red indicate increase or decrease in TG2 binding when mutated, respectively. Residues highlighted in blue comprise the N-X-S/T N-linked glycosylation sequence motif. Residues found mutated in human cancers annotated in the cBioPortal database are bolded and represented three-dimensionally in C. (B) Pairwise (mouse and human) surface conservation analysis of the conserved patch on the PLL domain to identify residues involved in TG2 binding. See Figure 2B. (C) GPR56 cancer mutations annotated in the cBioPortal database are mapped to the structure of the GPR56 ECR (PDB: 5KVM) and colored red. PLL domain, PLL-GAIN linker, and GAIN domain are colored cyan, pink, and gray, respectively. The cleaved C-terminal strand of the GAIN domain, termed *"Stachel"*, is colored dark green. All cysteine residues are colored yellow. See Figure 2.

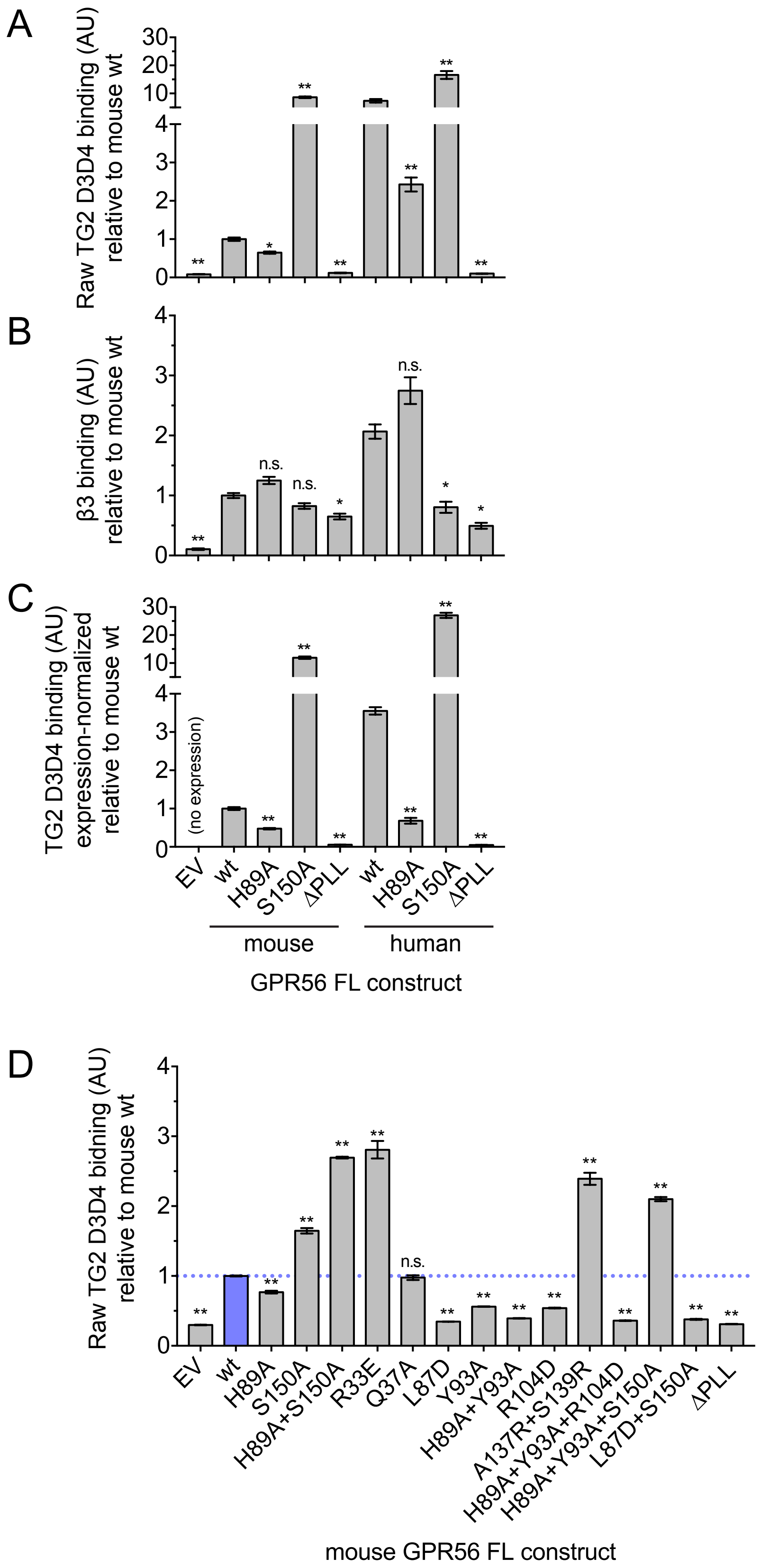

Salzman et al,. Figure S3

Figure S3. Differential cell surface expression and TG2 binding across GPR56 mutant constructs. (A-D) HEK293T cells transfected with indicated GPR56 FL construct were stained as outlined in Figure 2A. The bulk TG2 D3D4 binding signal (A and D), bulk cell-surface expression (B), and TG2 binding signal within a defined range of surface expression as outlined in Figure S4 (C) are presented. Binding signal represents MFI. Error bars indicate S.E.M. of n=3 independent measurements. Significance levels calculated by 2-way ANOVA with Bonferroni correction for multiple comparisons. Asterisks indicate comparison of mutant mouse and human GPR56 constructs with wt mouse and human GPR56, respectively. n.s., not significant; *$p<0.05$; **$p<0.001$. See Figure 2.

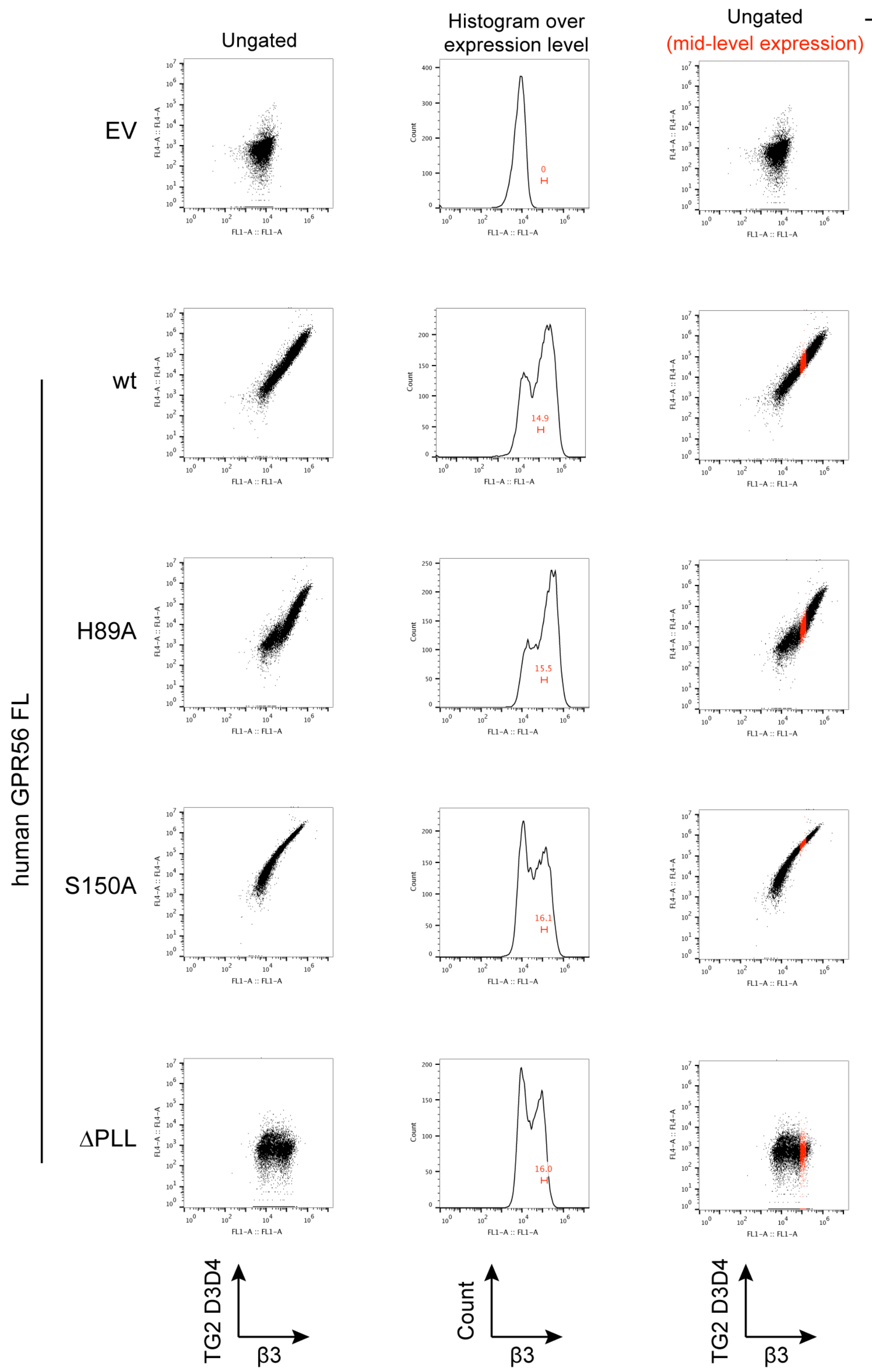

	"raw" β3 MFI (x10^4)	"raw" TG2 D3D4 MFI (x10^3)	"expression-normalized" TG2 D3D4 MFI (x10^3)
EV	0.8	0.6	N/A no expression
wt	12.4	46.8	44.1
H89A	14.3	12.3	8.9
S150A	3.6	85.5	346.9
ΔPLL	2.5	0.6	0.6

Salzman et al., Figure S4

Figure S4. Quantification of TG2 binding within a defined range of cell surface expression. Raw flow cytometry data from Figures S3A-C are displayed as a test case. “Raw” (i.e. bulk) TG2 MFI values are written in black in the table. To quantify relative TG2 binding signal between mutants with differential cell surface expression, a gate (red) was drawn to define a range of cell surface expression (e.g. mid-level expression). The cells included in this gate are colored red in the right column of dot plots. The “expression-normalized” TG2 MFI corresponds to the MFI of the red cells. This value (written in red in the table) represents expression-normalized TG2 D3D4 binding signal and is plotted on the y-axis in Figures 2D, 3B, S3D, and S6B. See Figures 2 and 3.

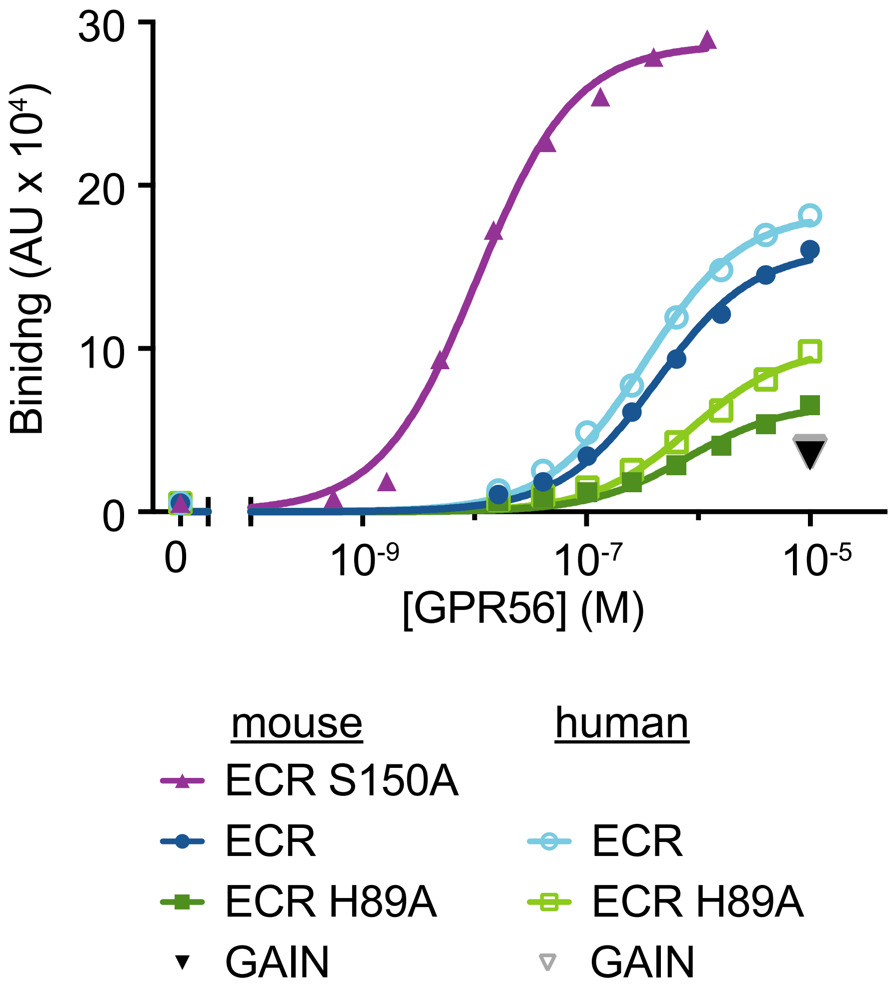

Figure S5. Binding affinity of TG2 for GPR56 is affected by mutations to the conserved patch on the PLL domain. Concentration titrations of monomeric GPR56 ECR constructs binding to mTG2 D3D4-coated M280 beads as represented in Figure 1B. GAIN domain is included as a control. Curves were fit to a simple one-to-one binding model to determine the dissociation constant, K_D, of each interaction: mouse ECR, 440 ± 20 nM; human ECR, 330 ± 15 nM; mouse ECR H89A, 880 ± 80 nM; human ECR H89A, 790 ± 11 nM; and mouse ECR S150A, 11 ± 0.7 nM. Binding signal represents MFI. Error bars indicate S.E.M. of n=3 independent measurements. See Figure 1.

A

	mouse GPR56			human GPR56		
Monobody	binds GAIN	binds PLL	blocks TG2	binds GAIN	binds PLL	blocks TG2
β1	X	X	X	✓		X
β3	✓	X	X	✓	X	X
β6	✓	X	X	✓	X	X
β7	X	X	X	X	✓	✓
β12	X	✓	✓	X	X	X

B

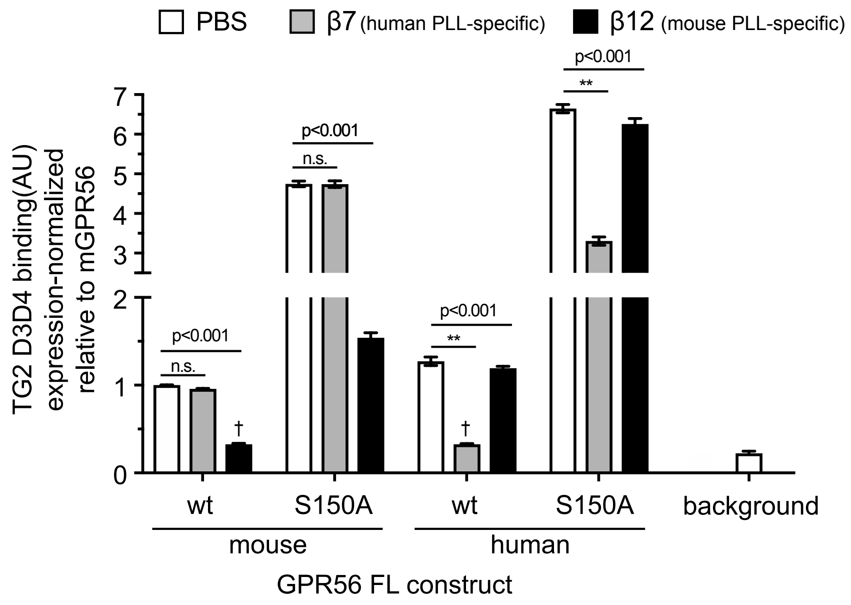

Figure S6. GPR56-binding monobody specificity and effect on TG2 binding. (A) Table indicating the minimum GPR56 fragment necessary and sufficient for binding of each monobody[2] as well as the observed effect on TG2 binding in the presence of excess monobody. (B) HEK293T cells transfected with indicated GPR56 FL constructs were stained with purified TG2 D3D4 in the presence of excess unlabeled monobody competitor as outlined in Figure 3A. Binding signal represents MFI. The “background” represents TG2 D3D4 binding signal to HEK293T cells not expressing GPR56. Error bars indicate S.E.M. of n=3 independent measurements. Significance levels shown were calculated by 2-way ANOVA with Bonferroni correction for multiple comparisons. n.s., not significant; †, not significant versus background. See Figure 3.

Supplementary References

1. Capra, J. A. & Singh, M. Predicting functionally important residues from sequence conservation. *Bioinformatics* **23**, 1875–1882 (2007).
2. Salzman, G. S. *et al.* Stachel-independent modulation of GPR56/ADGRG1 signaling by synthetic ligands directed to its extracellular region. *Proc. Natl. Acad. Sci. U. S. A.* **114**, 10095–10100 (2017).
